# Partial Nephrectomy in a Patient with a Left Ventricular Assist Device

**DOI:** 10.1155/2011/526903

**Published:** 2011-09-22

**Authors:** Jules P. Manger, John A. Kern, Tracey L. Krupski

**Affiliations:** ^1^Department of Urology, University of Virginia, Charlottesville, VA 22908, USA; ^2^Division of Thoracic and Cardiovascular Surgery, Department of Surgery, University of Virginia, Charlottesville, VA 22908, USA

## Abstract

Left ventricular assist device (LVAD) use has increased as a bridge to heart transplant as well as destination therapy in patients with severe heart failure. Presence of LVAD is not a contraindication to noncardiac surgery but does present special challenges to the surgical, anesthesia, and cardiac teams. We present the case of a 40-year-old woman with idiopathic cardiomyopathy necessitating LVAD who underwent left partial nephrectomy for a renal mass. She had undergone three nondiagnostic percutaneous image-guided biopsies. Left partial nephrectomy was performed. Perioperative care was without incident due to careful oversight by a multidisciplinary team. Pathology revealed high-grade clear cell renal cell carcinoma (RCC) with negative margins. Polytetrafluoroethylene (PTFE) bolsters were misidentified six months postoperatively on computed tomography (CT) at an outside institution as a retained laparotomy sponge. This is, to our knowledge, the first report of a partial nephrectomy performed in a patient with LVAD.

## 1. Introduction

Left ventricular assist device (LVAD) use has increased and become more successful and durable. It is the standard-of-care bridge to heart transplant in patients with severe cardiomyopathy [1]. It is also being used as destination therapy in some patients. Noncardiac surgery is complicated in LVAD due to the position of the device, need for anticoagulation, and tenuous hemodynamics of these patients. Nevertheless, successful noncardiac surgery in LVAD patients occurs when careful coordination between anesthetic, cardiac, and surgical teams takes place [2–4].

## 2. Case

**Figure 1:**
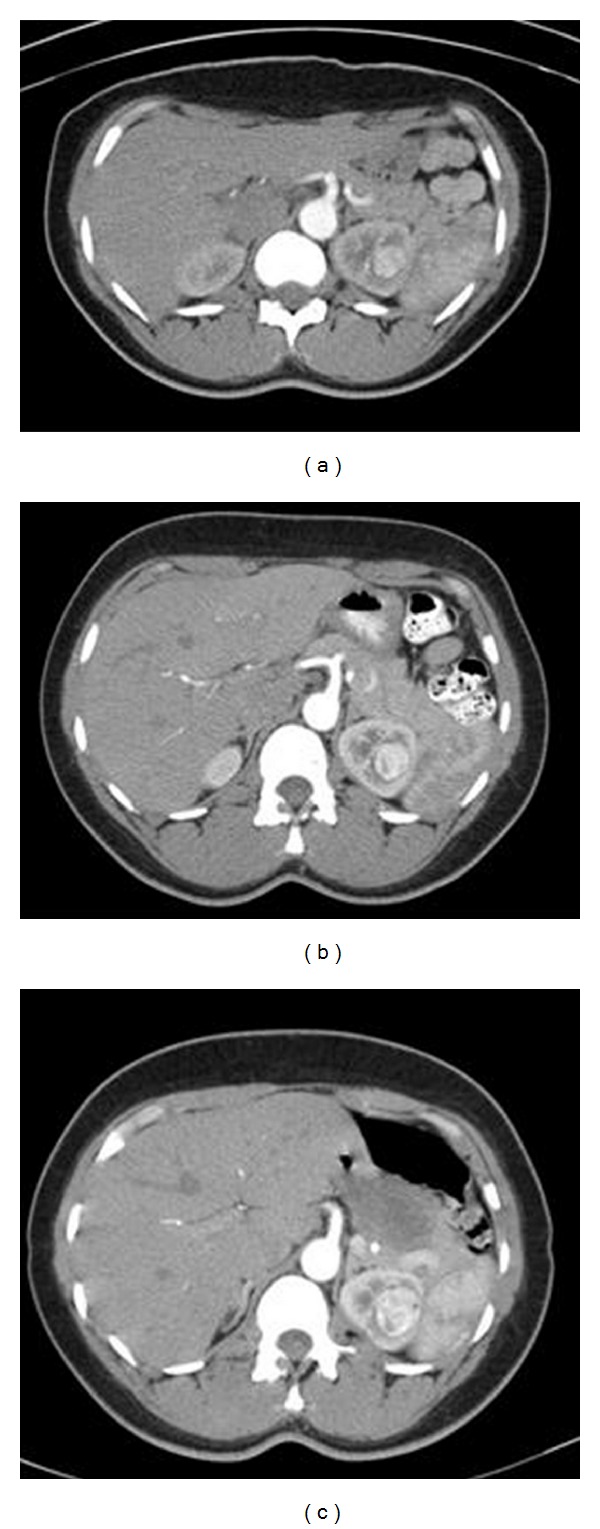
Serial contrasted CT scan of the abdomen demonstrating interval growth of enhancing left renal mass (12, 8, 4 months prior to partial nephrectomy).

A 40-year-old African-American female with a history of idiopathic familial cardiomyopathy was being evaluated for heart transplantation. Eleven months earlier, she had a CT abdomen performed as part of a cardiac transplant workup which revealed a 1.5 cm enhancing left upper pole renal mass (Figure 1). Given her age, it was possible that this lesion was a benign tumor such as angiomyolipoma or oncocytoma. Therefore, she underwent a CT-guided biopsy of this mass to reconcile the issue prior to heart transplant. This biopsy proved inconclusive due to insufficient tissue, and a second attempt was made under conscious sedation six months prior to LVAD placement. This attempt had to be aborted when she developed severe chest pain and dyspnea preventing adequate sampling. As her ejection fraction worsened, she became progressively dyspneic at rest and received a Heartmate II LVAD (Thoratec Corp., Pleasanton, CA). After much discussion of options, she underwent a CT-guided biopsy under general anesthesia which revealed oncocytic-type cells with fine needle aspiration but again was nondiagnostic. By the time the patient developed severe decompensated heart failure necessitating LVAD placement, the mass had grown to 2.7 cm (Figure 1). 

The patient had a past medical history of a nonischemic cardiomyopathy and resultant heart failure manifested by orthopnea, dyspnea on exertion, paroxysmal nocturnal dyspnea, palpitations, and angina. Transthoracic echocardiogram revealed a dilated and hypertrophic left ventricle with a left ventricular ejection fraction of 10–15% with no significant valvular pathology. Cardiac catheterization revealed the absence of coronary artery disease. She had undergone an implantable cardiac defibrillator due to her diminished ejection fraction, but the device had never discharged. 

Preoperatively, physical examination revealed a well-nourished, well-developed woman (70 kg, BMI 23.4) who was an active participant in decision making. She carried the LVAD in a satchel and had the expected vital signs for a patient with an LVAD. She had a well-healed scar over her sternum and upper abdomen. Trace pedal edema was present. Laboratory examination revealed hemoglobin of 9.2 g/dL, serum creatinine 0.9 mg/dL (estimated glomerular filtration rate 92 mL/min), international normalized ratio (INR) 2.6, and negative urine culture.

The decision was made to perform partial nephrectomy due to the imaging characteristics and growth rate of the mass and the imperative to clear her of a potential renal cell carcinoma in the setting of cardiac transplant listing. Given the need for future immunosuppression, the cardiologist, urologist, and cardiac surgeon felt that maximization of nephrons outweighed the risk of transient bleeding that may have been avoided by performing a radical nephrectomy. Percutaneous ablative approaches were not considered due to tumor location. The cardiology team assisted in medically maximizing the patient preoperatively. The patient was preadmitted to the urology service to facilitate the coordination of care. She was given a gentle bowel preparation of magnesium citrate along with one day of clear liquids. She was maintained off warfarin for 3 days preoperatively with therapeutic-dose enoxaparin which was discontinued the morning of surgery. Her INR on admission was 1.5. Anesthesiology was consulted, and the case was coordinated to ensure that a cardiac anesthesiologist administered general anesthesia. Additionally, the cardiac surgeon was available in the operating room the morning of the surgery. 

General anesthesia was initiated, and an arterial line was placed with the cardiac surgeon standing by. Extensive padding was utilized with respect to the LVAD as the patient was positioned in the left modified flank position. To avoid excessive pressure points, the kidney rest was not utilized. From a retroperitoneal approach just off the eleventh rib, the kidney was freed and the hilum dissected. At no point did the LVAD present any difficulties. The lesion was not readily identified on gross inspection. Intraoperative ultrasound revealed the mass in the superior pole abutting the collecting system. After injection of 6 mg of mannitol, the renal artery was clamped and then iced. After fifteen minutes of cooling, the capsule was scored with argon plasma coagulation and resection of the mass was performed with the blunt edge of a knife handle. Inferior and deep margins were sent for frozen examination and found to be negative for tumor. The collecting system was closed with 4–0 poliglecaprone suture. Argon plasma coagulation was performed to the resection bed. A Surgicel (Ethicon, Inc., Somerville, NJ) bolster and Avitene powder (Alcon, Inc., Humacao, Puerto Rico) were placed in the defect. The defect was closed with a 0-chromic suture on a liver needle bolstered with a strip of polytetrafluoroethylene (PTFE) pledget both anteriorly and posteriorly. Unclamping was performed for a total clamp time of 59 minutes, and the kidney was observed for 15 minutes. FloSeal (Baxter International Inc., Deerfield, IL) coagulation matrix was applied to the defect and hilum and argon plasma coagulation was performed on some oozing portions of Gerota's fascia. Gerota's fascia was then used to cover the kidney again. A 1.5 cm defect in the pleura was closed with 2–0 polyglactin, and the air was evacuated with a red rubber catheter. A Jackson-Pratt drain was left in the retroperitoneum to bulb suction, and the wound was closed. The patient was extubated and transferred to the cardiac care unit for postoperative care. The patient received at total of 3 units of packed red blood cells and one unit of fresh frozen plasma. Estimated blood loss was 500 mL.

Postoperative chest radiograph revealed no pneumothorax. Warfarin and enoxaparin were resumed on postoperative days two and four, respectively. Furosemide was restarted on postoperative day number one. The patient had her diet quickly advanced, and pain was controlled on oral narcotics. She was up and ambulatory, tolerating a low-sodium diet and had all of her heart failure medications restarted. Her Jackson-Pratt drain was removed on postoperative day number 4, and she went home on postoperative day number 6. Final pathology revealed clear cell RCC 2.6 × 2.0 × 1.5 cm with negative margins, Fuhrman grade 3 (high grade). The serum creatinine upon discharge was 0.8 mg/dL (eGFR 103 mL/min).

**Figure 2:**
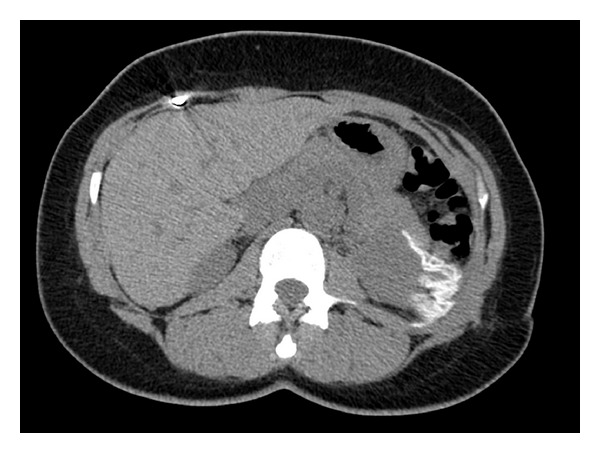
Postoperative CT abdomen demonstrating PTFE bolster on left kidney.

**Figure 3:**
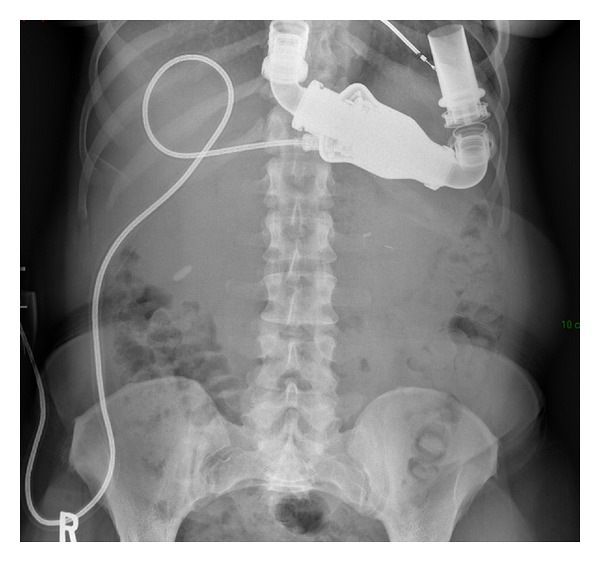
Confirmation of no retained sponge with series of abdominal plain films.

Her managed care consortium mandated postdischarge urologic care be performed at an outside institution. Her postoperative check was unremarkable, but at her 6 month followup she was experiencing worsening left flank pain. CT scan of the abdomen revealed a radiodense material over the superolateral aspect of the left kidney (Figure 2). The final impression stated that there was a retained laparotomy sponge, and the patient was informed that she would require further surgery. This distressed the patient, and she immediately wanted to return to the original surgeon, but the managed care consortium required prior approval which caused a 2-month delay in return to our clinic for evaluation. Upon reevaluation, plain abdominal radiographs were obtained in both the posterior-anterior and oblique orientations. This did not reveal ribbon-like radiopaque material which would be consistent with a retained laparotomy sponge (Figure 3). Reassurance was provided. 

## 3. Discussion

Noncardiac surgery can be safely and successfully performed in the setting of careful coordination between the surgical team and cardiology, cardiac anesthesiology, and cardiothoracic surgery teams. While nephrectomy [4] and pyelolithotomy [3] have been described in patients with LVAD, this is the first report to our knowledge of partial nephrectomy in such a patient. Management of renal cell carcinoma with nephron-sparing therapies has been increasing and is the standard of care in renal masses less than 4 cm. Nephron sparing is particularly crucial in patients with comorbidities which pose a long term threat to renal function such as heart disease, obesity, and diabetes mellitus. This patient had another future threat to her renal function in the form of calcineurin-inhibitor nephrotoxicity associated with heart transplantation. 

Communication was central to the success of this operative course. Preoperative evaluation by cardiology team and real-time consultations between urologist and anesthesiologist is critical to the success. The infrequent nature of concurrent malignancy in a heart transplant candidate with an LVAD means that there will never be a “process” of care pathway for these patients. Inclusion of this cardiac anesthesiologist was invaluable in terms of their experience with these patients. An arterial line was placed to monitor the pressure as noninvasive blood pressure monitoring is not reliable in patients with continuous flow LVAD. The blood bank was prepared for the case and the appropriate blood products were available for transfusion. Cardiothoracic surgery was aware and in the operative suite in the event of any complication with the device or questions regarding the surgical anatomy. Postoperative care was performed by the cardiac intensive care unit with the urology team as consultants. It is the practice at our institution for patients with LVAD to be admitted to the cardiology service because of the complexity of these patients. 

Careful surgical technique along with hemostatic agents and bolstered sutures enable partial nephrectomy to be performed without significant postoperative bleeding in most patients. Partial nephrectomy is high-risk surgery in patients requiring chronic anticoagulation due to both bleeding [5] and thrombotic [6] complications. In fact, at our institution, initiation of anticoagulation (even prophylactic dose) or antiplatelet agents is avoided after partial nephrectomy. Due to the fact that the patient had a HeartMate II device rather than pulsatile flow device, there was no necessity of immediate postoperative anticoagulation [7]. Nevertheless, reinstitution of warfarin and enoxaparin as soon as surgically safe was recommended. 

An interesting aspect of this case was the failure to diagnose renal cell carcinoma preoperatively. It is not our practice to biopsy enhancing renal masses prior to performing partial nephrectomy. However, in the setting of young age, chronic anticoagulation, and severe heart failure, tissue diagnosis was sought. On three separate occasions, percutaneous CT-guided biopsy of the mass failed to yield a diagnosis. We often perform renal biopsy in concert with ablative therapies but rarely biopsy renal masses prior to surgery. This case illustrates one of the common pitfalls with percutaneous renal biopsy which is inability to obtain an accurate diagnosis [8]. 

The reason that partial nephrectomy was performed in this high-risk patient without tissue diagnosis is that she was on the heart transplant list. Search for malignancy is performed prior to heart transplant. Patients will be deprioritized until these issues are resolved. One of the most common causes of late death in cardiac transplant is immunosuppression-driven malignancy. Retrospective studies and animal models have demonstrated that immunosuppression is not only a known risk of de novo malignancies but also fuels recurrence, progression, and metastasis of known or even treated lesions [9–13]. 

The renal capsule and parenchymal defect was reapproximated in this case with hemostatic suture utilizing PTFE pledgets to redistribute the forces of the suture and prevent tearing of the renal capsule and kidney parenchyma. Such a large strip would not usually be used, but this patient had moderate blood loss due to both the position of the tumor and her chronic anticoagulation. This increased volume of PTFE was needed to secure hemostasis. PTFE is a material with broad applications in cardiac, vascular, and general surgery. It is radiodense on CT but not radiopaque on plain film. Its use in partial nephrectomy has been described in the literature [14, 15] as well as its radiologic characteristics on CT [16]. However, its application in partial nephrectomy and imaging characteristics are not well known by the medical community. We have observed several outside radiologists who misidentify PTFE pledgets as retained laparotomy sponge. This causes a great deal of distress and anxiety to the patient and yields unnecessary testing and cost as evidenced by this case. 

We present the case of a partial nephrectomy performed in a 40-year-old woman with idiopathic cardiomyopathy. Noncardiac surgeries, even those with a high risk of bleeding complications, may be performed in the setting of LVAD as a multidisciplinary effort at a tertiary care center. Principles of surgery in such patients include the following:

- communication between cardiology, cardiac anesthesia, cardiothoracic surgery, and the primary surgical team;
- thoughtful perioperative anticoagulation with less stringent requirement in continuous flow devices;
- arterial line for hemodynamic monitoring particularly in continuous flow devices;
- careful positioning and avoidance of the device and drivelines;
- generous use of hemostatic techniques and agents.

This case illustrates the imperative for diagnosis and treatment of suspicious renal mass in potential transplant recipients since immunosuppression drives tumorigenesis and tumor growth in many malignancies including RCC. This is a case of unsatisfying results from percutaneous biopsy of a renal mass. Finally, highlighted is the lack of awareness of PTFE use in partial nephrectomy and its radiographic characteristics.
